# Function of G-Protein-Coupled Estrogen Receptor-1 in Reproductive System Tumors

**DOI:** 10.1155/2016/7128702

**Published:** 2016-05-29

**Authors:** Hongyan Qian, Jingxiu Xuan, Yuan Liu, Guixiu Shi

**Affiliations:** Department of Rheumatology and Clinical Immunology, The First Affiliated Hospital of Xiamen University, Xiamen 361003, China

## Abstract

The G-protein-coupled estrogen receptor-1 (GPER-1), also known as GPR30, is a novel estrogen receptor mediating estrogen receptor signaling in multiple cell types. The progress of estrogen-related cancer is promoted by GPER-1 activation through mitogen-activated protein kinases (MAPK), phosphoinositide 3-kinase (PI3K), and phospholipase C (PLC) signaling pathways. However, this promoting effect of GPER-1 is nonclassic estrogen receptor (ER) dependent manner. In addition, clinical evidences revealed that GPER-1 is associated with estrogen resistance in estrogen-related cancer patients. These give a hint that GPER-1 may be a novel therapeutic target for the estrogen-related cancers. However, preclinical studies also found that GPER-1 activation of its special agonist G-1 inhibits cancer cell proliferation. This review aims to summarize the characteristics and complex functions of GPER-1 in cancers.

## 1. Introduction

Estrogen is an important hormone in human beings, especially in females. It plays several important physiological and pathological roles in not only reproductive system but also other systems. Estrogen disorder results in various diseases, such as endometrial diseases, skeletal diseases, and reproductive system tumors. Increasing attention has been paid to revealing of the functions of estrogen in physiological and pathological conditions. Estrogen receptors (ER) *α* and *β*, the two well established nuclear estrogen receptors, have different physiological functions depending upon their various distributions [1]. Meanwhile, the activity of ER*β* is opposed to ER*α* in many systems. Lots of evidences show that estrogen induces the proliferation of cancer cells in breast, uterus, and ovarian cancer through ER*α*. On the contrary, activation of ER*β* can reverse this effect. Notably, a novel transmembrane estrogen receptor, known as G-couple estrogen receptor (GPER), was found [2]. ER are involved in the initiation, migration, and progression of estrogen-related multiorgan cancers, such as breast cancer, ovarian cancer, prostate cancer, testicular cancer, liver cancer, and lung cancer as well [3]. Although increasing studied are focused on the roles of GPER-1 in different types of cancers, the functions of GPER-1 in cancers remain unclear yet. Characteristics and functions of reproductive system cancer will be summarized and discussed in the present review.

## 2. The Characteristics of GPER-1

### 2.1. The Structure and Distribution of GPER-1

G-protein-coupled estrogen receptor-1 (GPER-1), a seven-transmembrane-domain receptor localized in cell surface, was first identified in 1996 [2]. GPER-1 is detected broadly in numerous human tissues, such as breast, prostate, ovary, placenta, subcutaneous adipose, visceral adipose, arteries, vessels, heart, liver, lung and intestine tissues. GPER-1 is a member of GPCR superfamily, which is structurally unrelated to the classical ER*α* and ER*β*. There are four transcriptional variants encoding 375 amino acids composing seven transmembrane proteins [4].

Classical GPCR are cell membrane proteins which bind their ligands at cell surface. But GPER-1 binding domain exists inside the plasma membranes and the endoplasmic reticulum [5–8]. The biological functions of GPER-1 might be associated with cell types and its location. Estradiol, the major type of estrogen, binds to GPER-1 with a high affinity to GPER-1 while the other two isoforms, estrone and estriol, have very low binding affinities [5, 9]. Furthermore, numbers of environmental estrogen bind to GPER-1 and activate the downstream signaling pathways, such as bisphenol A, genistein, and nonylphenol [10]. GPER-1-specific compound 1 (G-1) is a specific agonist of GPER-1, which has no function of ER*α* and ER*β* and was identified using virtual and biomolecular screening in 2006 [11]. G-1 has been widely used as a target tool to evaluate the function of GPER-1 in different cells and disease models.

### 2.2. GPER-1 Signaling Pathways

GPER-1 mediates both genomic and nongenomic response with its ligands. To date, GPER-1 signaling pathways have not been fully elucidated yet. The binding ligands of GPER-1, such as estrogen, G-1, tamoxifen, and ICI182,780, cross the plasma membrane and bind to the GPER-1 on endoplasmic reticulum where they activate its *β* and *γ* subunits and subsequently activate both Src and adenylyl cyclase (AC) leading to the intracellular cAMP production. The phosphorylation of Src induces matrix metalloproteinase (MMP) production, which cleaves pro-heparan-bound epidermal growth factor (pro-HB-EGF) releasing free HB-EGF. HB-EGF binds to the EGFR leading to activation of multiple molecules such as Ras, PI3K, AKT, and Erk1/2. The downstream signal of PI3K and AKT results in several nuclear receptors activation which is closely related the proliferation and migration of cancer cell. GPER-1 also binds to the G-couple protein *α* subunit and activates the phospholipase C (PLC), AC, and CAMP. Activated PLC results in inositol triphosphate (IP3) production, which further binds to its receptor and leads to intracellular calcium mobilization.

## 3. Functions of GPER-1 in Reproductive System Tumors

### 3.1. Breast Cancer

Breast cancer is the most common and deadly cancer in females worldwide [12]. Breast cancer is generally classified into estrogen receptor positive (ER+) and ER negative (ER−) [13]. In clinical practices, the endocrine treatments such as tamoxifen and aromatase are recommended in the ER positive breast cancers, while there is no benefit in the ER negative cancers [14]. GPER-1 is widely expressed in both of these breast cancer types and the primary breast cancers. Recent clinical study results showed that the expression of GPER-1 might correlate with clinical and pathological poor outcome biomarkers [15]. Other results also showed that the expression of GPER-1 was inversely correlated with the ER expression. Coexpression of GPER-1 and ER was found in almost 24% patients with inflammatory breast cancer, while 19% only express ER and 46% only express GPER-1 [16].

The GPER-1 mRNA levels were significant higher in ER positive breast cancer cells compared to ER negative cancer cells, and the expression of GPER-1 depends on ER*α* mRNA level. Interestingly, GPER-1 preformed a different proliferation manner in ER positive MCF-7 breast cancer cell line [17]. G-1 enhanced migration of MCF-7 breast cancer cells by activating ERK1/2 and EGFR signaling pathway, which is tremendously attenuated by G15 [18]. The other evidences also approved that GPER-1 is an initiator of tamoxifen resistance in breast cancers [19–21]. The promotion roles of GPER-1 in cancer cells proliferation and migration may be correlated with the autolysis of calpain 1 [22], cleavage of cyclin E [18], or the expression of target gene. There are also some studies which found that GPER-1 inhibits the growth of ER positive MCF-7 cells, which is probably through G-couple *β* and *γ* subunits activating without CAMP signal activation [21–24]. However, combination treatment with G-1 and Her2 antibody Trastuzumab exerted an additive growth inhibitory effect on breast cancer cells [25]. Thus, GPER-1 inhibits ER positive breast cancers proliferation which is a potential target for ER positive breast cancers and drug-resistant breast cancer.

ER negative breast cancer cells are more aggressive than ER positive cancer cells. Deficiency of ER in breast cancer is correlated with poor response to endocrine therapy [26]. In ER negative breast cancers, GPER-1 stimulates the ERK1/2 through the EGFR/MAPK signal cascade, inducing target gene like c-fos expression, which is involved in the progressing of breast malignancies [27–29]. Estrogen and antiestrogens can also promote the production of the early growth response-1 (Egr-1), connective tissue growth factor (CTGF), and insulin-like growth factor 1 (IGF-1) through the GPER-1 [28, 30, 31]. GRP30 activation stimulated breast cancer cells migration through CTGH, CXC receptor (CXCR1), and notch pathways [28]. Furthermore, GPER-1 agonist G-1 promoted inflammation in breast cancers [32]. GPER-1 was reported to affect the deformation of breast glandular structure inducing the malignant transformation of breast tissue [33]. GPER-1 can also induce expression of cancer-associated fibroblasts (CAFs) in tumor microenvironment [34, 35]. On the contrary, a recent study showed that activation of GPER-1 by G-1 resulted in G2/M-phase arrest and induction of mitochondrial-related apoptosis [36]. The other studies also proved that G-1 treatment suppressed the growth of SKBr3 cancer cells and increased the survival rate by inducing the ERK1/2 signal activation [36, 37].

15–20% of breast cancers are included in triple negative breast cancers (TNBC), characterized by lack of ER*α*, progesterone receptor (PR), and EGFR2 (Her-2). A higher rate of recurrence and aggressive biological features were found in younger females [38, 39]. GPER-1 expression was found in majority of TNBCs patients [40]. In the GPER knockdown mice model, the proliferation of TNBCs, the activation of EGFR, and c-fos expression were reduced [41]. These findings suggest that GPER plays a key role in putative mechanism for TNBCs and GPER might be a therapeutic target for TNBCs.

Paradoxical debates still exist on the functions of GPER-1 in breast cancers. SNPs of GPER-1, histone acetylation, and transcription factor recruitment were significantly associated with tumor size and histological grading [42, 43]. The different results of GPER-1 in breast cancer were summarized in Table 1.

### 3.2. Ovarian Tumors

Estrogens play a crucial role in the development of ovarian cancers. GPER RNA as well as GPER-1 protein presents in both primary and malignant ovarian tumor tissues [44]. The expression of GPER-1 was significantly increased in ovarian carcinomas compared to pericarcinomatous tissues impendent with the expression of EGFR, ER*α*, and ER*β* [45]. Further investigation showed that the expression of GPER-1 was associated with lower survival rates [46]. Estrogen and G-1 induce ovarian cancer cell growth responses via EGFR-MAPK signaling pathways. This procedure required coexpression of ER*α* [44, 47]. Furthermore, GPER-1 promoted the migration and invasion of ovarian cancer cells OVCAR5 which is characterized by negative ER*α* and positive GPER by increasing the expression MMP-9 [48, 49]. Atrazine, one of the most common pesticide contaminants, promoted ovarian cancer cells proliferation via induction of Erk and expression of estrogen target gene through GPER-1 pathway [50]. But other studies results showed that G-1 suppressed proliferation and induced apoptosis of human ovarian cancer cells probably through inhibition of cell cycle progression in G2/M-phase in ovarian carcinomas [51, 52].

### 3.3. Testicular Cancers

GPER-1 has been shown to be involved in a variety of hormone-dependent cancers. It is well understood that estrogens play a critical role in pathological germ cell proliferation in testicular germ cell tumors. GPER-1 seems to be involved in modulating the growth of estrogen dependent testicular cancer cells [53]. Estrogen induces the high expression of GPER-1 correlated with low levels of ER*β* in human testicular carcinoma in situ and seminomas [53, 54]. Bisphenol A, a common environmental estrogen, can also promote the proliferation of testicular seminomas cells through GPER-1 [55]. The above findings suggested that GPER-1 may be a potential therapeutic target [56, 57].

### 3.4. Prostate Cancer

Estrogen has an efficacy for advanced prostate cancer (PC) via the mediation of the classical estrogen receptors [58]. The effects of ER on PC growth and metastases have different mechanisms in different cellular microenvironments [59]. The expression of GPER-1 is higher in the preneoplastic lesions and normal areas of benign prostate than the basal epithelial cells [60]. G-1, the selectively activating GPER-1, inhibited the growth of multiple PC cells in vitro and in vivo through Erk1/2 and c-jun/c-fos signaling pathways, which indicates that the G-1 may be a new option for PC through targeting GPER-1 [61]. G-1 inhibited castration-resistant phase but had no effect on androgen-sensitive tumors. The antitumor effect of G-1 on CR tumors was related to necrosis (approximately 65%) accompanied with neutrophils infiltration. G-1 can also upregulate neutrophil-related chemokines and inflammation-mediated cytokines in the CR tumors. In one word, GPER-1 is an androgen-repressed target. The antitumor effect of G-1 was neutrophil-infiltration-associated necrosis [62].

## 4. GPER-1 in Other Tumors

Overexpression of GPER-1 was detected in various reproductive system cancers. Studies showed that the activation of GPER-1 signaling pathways leads to tumor. There are other studies which proved that GPER-1 induced proliferation, differentiation, and drug resistance of lung cancers [63, 64], thyroid cancers [65], bladder cancers [66], and oral squamous carcinomas [67]. More studies to reveal the functions and mechanisms of GPER-1 in the other system cancers are warranted.

## 5. Conclusion

GPER-1 activation by estrogen induces nongenomic signaling pathways and regulates certain gene transcriptions. Majority of the study results addressed that activation of GPER-1 by estrogen and G-1 results in the downstream signals and target genes activation, which promotes the proliferation, migration, and invasion of cancer cells. And this effect is in nonclassical ER expression dependent manner in most cancers except for ovarian cancers. It is interesting that several other studies showed that G-1, the special agonist of GPER-1, promoted the expression of GPER-1 and inhibited the proliferation of ER negative breast cancer cells, ovarian cancer cells, and prostate cancer cells. The opposite effects of GPER-1 in cancer cells may be associated with the epigenetic of GPER-1, such as the SNPs and histone acetylation. The different cell types, tumor microenvironment, and hormonal level may also affect the functions of GPER-1. Controversies still exist on the GPER-1 localization and related signaling pathways, in particular the potential action as proapoptotic mediator. Since the function and mechanisms of GPER-1 are still unclear, more researches and clinical studies are strongly warranted to clarify the different function and mechanisms in different cancer types and conditions.

## Figures and Tables

**Table 1:** The effect of GPER-1 in reproductive system tumors.

Cancer types	ER positive breast cancer	ER negative breast cancer	Triple negative breast cancer	Ovarian cancer	Testicular cancer	Prostate cancer
Proliferation	+24, 31	+28, 31	+40, 41	+47, 48, 49, 50	+55, 56, 57	−61, 62
	−17, 25, 52	−23, 36, 37		−51, 52		

Migration and metastasis	+18, 21, 22, 31, 15, 32	+27, 28, 29	+41	+48, 49	/	/

Poor survival	+20, 16	−36	+38, 39, 40	+46	/	/

Drug resistance	+19, 20, 21	+26	/	/	/	/

## References

[1] Dechering K., Boersma C., Mosselman S. (2000). Estrogen receptors *α* and *β*: two receptors of a kind?. *Current Medicinal Chemistry*.

[2] Owman C., Blay P., Nilsson C., Lolait S. J. (1996). Cloning of human cDNA encoding a novel heptahelix receptor expressed in Burkitt's lymphoma and widely distributed in brain and peripheral tissues. *Biochemical and Biophysical Research Communications*.

[3] Chen G. G., Zeng Q., Tse G. M. K. (2008). Estrogen and its receptors in cancer. *Medicinal Research Reviews*.

[4] Wang C., Prossnitz E. R., Roy S. K. (2007). Expression of G protein-coupled receptor 30 in the hamster ovary: differential regulation by gonadotropins and steroid hormones. *Endocrinology*.

[5] Thomas P., Pang Y., Filardo E. J., Dong J. (2005). Identity of an estrogen membrane receptor coupled to a G protein in human breast cancer cells. *Endocrinology*.

[6] Funakoshi T., Yanai A., Shinoda K., Kawano M. M., Mizukami Y. (2006). G protein-coupled receptor 30 is an estrogen receptor in the plasma membrane. *Biochemical and Biophysical Research Communications*.

[7] Revankar C. M., Cimino D. F., Sklar L. A., Arterburn J. B., Prossnitz E. R. (2005). A transmembrane intracellular estrogen receptor mediates rapid cell signaling. *Science*.

[8] Revankar C. M., Mitchell H. D., Field A. S. (2007). Synthetic estrogen derivatives demonstrate the functionality of intracellular GPR30. *ACS Chemical Biology*.

[9] Prossnitz E. R., Oprea T. I., Sklar L. A., Arterburn J. B. (2008). The ins and outs of GPR30: a transmembrane estrogen receptor. *Journal of Steroid Biochemistry and Molecular Biology*.

[10] Thomas P., Dong J. (2006). Binding and activation of the seven-transmembrane estrogen receptor GPR30 by environmental estrogens: a potential novel mechanism of endocrine disruption. *Journal of Steroid Biochemistry and Molecular Biology*.

[11] Bologa C. G., Revankar C. M., Young S. M. (2006). Virtual and biomolecular screening converge on a selective agonist for GPR30. *Nature Chemical Biology*.

[12] DeSantis C., Ma J., Bryan L., Jemal A. (2014). Breast cancer statistics, 2013. *CA: A Cancer Journal for Clinicians*.

[13] Deblois G., Giguère V. (2013). Oestrogen-related receptors in breast cancer: control of cellular metabolism and beyond. *Nature Reviews Cancer*.

[14] Davies C., Godwin J., Gray R. (2011). Relevance of breast cancer hormone receptors and other factors to the efficacy of adjuvant tamoxifen: patient-level meta-analysis of randomised trials. *The Lancet*.

[15] Filardo E. J., Graeber C. T., Quinn J. A. (2006). Distribution of GPR30, a seven membrane-spanning estrogen receptor, in primary breast cancer and its association with clinicopathologic determinants of tumor progression. *Clinical Cancer Research*.

[16] Arias-Pulido H., Royce M., Gong Y. (2010). GPR30 and estrogen receptor expression: new insights into hormone dependence of inflammatory breast cancer. *Breast Cancer Research and Treatment*.

[17] Ahola T. M., Manninen T., Alkio N., Ylikomi T. (2002). G protein-coupled receptor 30 is critical for a progestin-induced growth inhibition in MCF-7 breast cancer cells. *Endocrinology*.

[18] Li Y., Chen Y., Zhu Z.-X. (2013). 4-Hydroxytamoxifen-stimulated processing of cyclin E is mediated via G protein-coupled receptor 30 (GPR30) and accompanied by enhanced migration in MCF-7 breast cancer cells. *Toxicology*.

[19] Mo Z., Liu M., Yang F. (2013). GPR30 as an initiator of tamoxifen resistance in hormone-dependent breast cancer. *Breast Cancer Research*.

[20] Ignatov A., Ignatov T., Weienborn C. (2011). G-protein-coupled estrogen receptor GPR30 and tamoxifen resistance in breast cancer. *Breast Cancer Research and Treatment*.

[21] Ignatov A., Ignatov T., Roessner A., Costa S. D., Kalinski T. (2010). Role of GPR30 in the mechanisms of tamoxifen resistance in breast cancer MCF-7 cells. *Breast Cancer Research and Treatment*.

[22] Chen Y., Li Z., He Y. (2014). Estrogen and pure antiestrogen fulvestrant (ICI 182 780) augment cell-matrigel adhesion of MCF-7 breast cancer cells through a novel G protein coupled estrogen receptor (GPR30)-to-calpain signaling axis. *Toxicology and Applied Pharmacology*.

[23] Ariazi E. A., Brailoiu E., Yerrum S. (2010). The G protein-coupled receptor GPR30 inhibits proliferation of estrogen receptor-positive breast cancer cells. *Cancer Research*.

[24] Wróbel A. M., Gregoraszczuk E. Ł. (2015). Action of methyl-, propyl- and butylparaben on GPR30 gene and protein expression, cAMP levels and activation of ERK1/2 and PI3K/Akt signaling pathways in MCF-7 breast cancer cells and MCF-10A non-transformed breast epithelial cells. *Toxicology Letters*.

[25] Lubig J., Lattrich C., Springwald A. (2012). Effects of a combined treatment with GPR30 agonist G-1 and herceptin on growth and gene expression of human breast cancer cell lines. *Cancer Investigation*.

[26] Ruan S.-Q., Wang Z.-H., Wang S.-W. (2012). Heregulin-*β*1-induced GPR30 upregulation promotes the migration and invasion potential of SkBr3 breast cancer cells via ErbB2/ErbB3-MAPK/ERK pathway. *Biochemical and Biophysical Research Communications*.

[27] Maggiolini M., Vivacqua A., Fasanella G. (2004). The G protein-coupled receptor GPR30 Mediates c-fos up-regulation by 17*β*-estradiol and phytoestrogens in breast cancer cells. *The Journal of Biological Chemistry*.

[28] Pandey D. P., Lappano R., Albanito L., Madeo A., Maggiolini M., Picard D. (2009). Estrogenic GPR30 signalling induces proliferation and migration of breast cancer cells through CTGF. *The EMBO Journal*.

[29] Filardo E. J., Quinn J. A., Bland K. I., Frackelton A. R. (2000). Estrogen-induced activation of Erk-1 and Erk-2 requires the G protein-coupled receptor homolog, GPR30, and occurs via trans-activation of the epidermal growth factor receptor through release of HB-EGF. *Molecular Endocrinology*.

[30] Vivacqua A., Romeo E., De Marco P., De Francesco E. M., Abonante S., Maggiolini M. (2012). GPER mediates the Egr-1 expression induced by 17*β*-estradiol and 4-hydroxitamoxifen in breast and endometrial cancer cells. *Breast Cancer Research and Treatment*.

[31] De Marco P., Bartella V., Vivacqua A. (2013). Insulin-like growth factor-I regulates GPER expression and function in cancer cells. *Oncogene*.

[32] Ohshiro K., Schwartz A. M., Levine P. H., Kumar R. (2012). Alternate estrogen receptors promote invasion of inflammatory breast cancer cells via non-genomic signaling. *PLoS ONE*.

[33] Marchese S., Silva E. (2012). Disruption of 3D MCF-12A breast cell cultures by estrogens—an in vitro model for ER-mediated changes indicative of hormonal carcinogenesis. *PLoS ONE*.

[34] Pupo M., Vivacqua A., Perrotta I. (2013). The nuclear localization signal is required for nuclear GPER translocation and function in breast Cancer-Associated Fibroblasts (CAFs). *Molecular and Cellular Endocrinology*.

[35] Santolla M. F., Lappano R., De Marco P. (2012). G protein-coupled estrogen receptor mediates the Up-regulation of fatty acid synthase induced by 17*β*-estradiol in cancer cells and cancer-associated fibroblasts. *The Journal of Biological Chemistry*.

[36] Wei W., Chen Z.-J., Zhang K.-S. (2014). The activation of G protein-coupled receptor 30 (GPR30) inhibits proliferation of estrogen receptor-negative breast cancer cells *in vitro* and *in vivo*. *Cell Death and Disease*.

[37] Chimento A., Casaburi I., Rosano C. (2014). Oleuropein and hydroxytyrosol activate GPER/ GPR30-dependent pathways leading to apoptosis of ER-negative SKBR3 breast cancer cells. *Molecular Nutrition and Food Research*.

[38] Venkitaraman R. (2010). Triple-negative/basal-like breast cancer: clinical, pathologic and molecular features. *Expert Review of Anticancer Therapy*.

[39] Chen J.-Q., Russo J. (2009). ER*α*-negative and triple negative breast cancer: molecular features and potential therapeutic approaches. *Biochimica et Biophysica Acta*.

[40] Steiman J., Peralta E. A., Louis S., Kamel O. (2013). Biology of the estrogen receptor, GPR30, in triple negative breast cancer. *The American Journal of Surgery*.

[41] Girgert R., Emons G., Gründker C. (2012). Inactivation of GPR30 reduces growth of triple-negative breast cancer cells: possible application in targeted therapy. *Breast Cancer Research and Treatment*.

[42] Giess M., Lattrich C., Springwald A., Goerse R., Ortmann O., Treeck O. (2010). GPR30 gene polymorphisms are associated with progesterone receptor status and histopathological characteristics of breast cancer patients. *Journal of Steroid Biochemistry and Molecular Biology*.

[43] Li Y., Birnbaumer L., Teng C. T. (2010). Regulation of ERR*α* gene expression by estrogen receptor agonists and antagonists in SKBR3 breast cancer cells: differential molecular mechanisms mediated by G protein-coupled receptor GPR30/GPER-1. *Molecular Endocrinology*.

[44] Kolkova Z., Casslén V., Henic E. (2012). The G protein-coupled estrogen receptor 1 (GPER/GPR30) does not predict survival in patients with ovarian cancer. *Journal of Ovarian Research*.

[45] Fujiwara S., Terai Y., Kawaguchi H. (2012). GPR30 regulates the EGFR-Akt cascade and predicts lower survival in patients with ovarian cancer. *Journal of Ovarian Research*.

[46] Smith H. O., Arias-Pulido H., Kuo D. Y. (2009). GPR30 predicts poor survival for ovarian cancer. *Gynecologic Oncology*.

[47] Albanito L., Madeo A., Lappano R. (2007). G protein-coupled receptor 30 (GPR30) mediates gene expression changes and growth response to 17*β*-estradiol and selective GPR30 ligand G-1 in ovarian cancer cells. *Cancer Research*.

[48] Yan Y., Liu H., Wen H. (2013). The novel estrogen receptor GPER regulates the migration and invasion of ovarian cancer cells. *Molecular and Cellular Biochemistry*.

[49] Yan Y., Jiang X., Zhao Y., Wen H., Liu G. (2015). Role of GPER on proliferation, migration and invasion in ligand-independent manner in human ovarian cancer cell line SKOV3. *Cell Biochemistry and Function*.

[50] Albanito L., Lappano R., Madeo A. (2015). Effects of atrazine on estrogen receptor *α*– and G protein–coupled receptor 30–mediated signaling and proliferation in cancer cells and cancer-associated fibroblasts. *Environmental Health Perspectives*.

[51] Ignatov T., Modl S., Thulig M. (2013). GPER-1 acts as a tumor suppressor in ovarian cancer. *Journal of Ovarian Research*.

[52] Wang C., Lv X., Jiang C., Davis J. S. (2012). The putative G-protein coupled estrogen receptor agonist G-1 suppresses proliferation of ovarian and breast cancer cells in a GPER-independent manner. *American Journal of Translational Research*.

[53] Chimento A., Sirianni R., Casaburi I., Pezzi V. (2014). GPER signaling in spermatogenesis and testicular tumors. *Frontiers in Endocrinology*.

[54] Boscia F., Passaro C., Gigantino V. (2015). High levels of GPR30 protein in human testicular carcinoma in situ and seminomas correlate with low levels of estrogen receptor-beta and indicate a switch in estrogen responsiveness. *Journal of Cellular Physiology*.

[55] Chevalier N., Bouskine A., Fenichel P. (2012). Bisphenol A promotes testicular seminoma cell proliferation through GPER/GPR30. *International Journal of Cancer*.

[56] Sandner F., Welter H., Schwarzer J. U., Köhn F. M., Urbanski H. F., Mayerhofer A. (2014). Expression of the oestrogen receptor GPER by testicular peritubular cells is linked to sexual maturation and male fertility. *Andrology*.

[57] Vitale G., Dicitore A., Sciortino G., Abbruzzese A., Persani L., Santini D. (2011). Role of gpr30 in testicular germ cell tumors: a potential new anticancer target. *Cancer Biology and Therapy*.

[58] Oh W. K. (2002). The evolving role of estrogen therapy in prostate cancer. *Clinical Prostate Cancer*.

[59] Claessens F., Tilley W. (2014). Androgen signalling and steroid receptor crosstalk in endocrine cancers. *Endocrine-Related Cancer*.

[60] Rago V., Romeo F., Giordano F., Ferraro A., Carpino A. (2016). Identification of the G protein-coupled estrogen receptor (GPER) in human prostate: expression site of the estrogen receptor in the benign and neoplastic gland. *Andrology*.

[61] Chan Q. K. Y., Lam H.-M., Ng C.-F. (2010). Activation of GPR30 inhibits the growth of prostate cancer cells through sustained activation of Erk1/2, c-jun/c-fos-dependent upregulation of p21, and induction of G2 cell-cycle arrest. *Cell Death and Differentiation*.

[62] Lam H.-M., Ouyang B., Chen J. (2014). Targeting GPR30 with G-1: a new therapeutic target for castration-resistant prostate cancer. *Endocrine-Related Cancer*.

[63] Jala V. R., Radde B. N., Haribabu B., Klinge C. M. (2012). Enhanced expression of G-protein coupled estrogen receptor (GPER/GPR30) in lung cancer. *BMC Cancer*.

[64] Zhang K.-S., Chen H.-Q., Chen Y.-S. (2014). Bisphenol A stimulates human lung cancer cell migration via upregulation of matrix metalloproteinases by GPER/EGFR/ERK1/2 signal pathway. *Biomedicine and Pharmacotherapy*.

[65] Vivacqua A., Bonofiglio D., Albanito L. (2006). 17*β*-Estradiol, genistein, and 4-hydroxytamoxifen induce the proliferation of thyroid cancer cells through the G protein-coupled receptor GPR30. *Molecular Pharmacology*.

[66] Huang W., Chen Y., Liu Y. (2015). Roles of ER *β* and GPR30 in proliferative response of human bladder cancer cell to estrogen. *BioMed Research International*.

[67] Bai L.-Y., Weng J.-R., Hu J.-L., Wang D., Sargeant A. M., Chiu C.-F. (2013). G15, a GPR30 antagonist, induces apoptosis and autophagy in human oral squamous carcinoma cells. *Chemico-Biological Interactions*.

